# Silver nanoparticles exert concentration‐dependent influences on biofilm development and architecture

**DOI:** 10.1111/cpr.12616

**Published:** 2019-05-03

**Authors:** Jingyang Guo, Simin Qin, Yan Wei, Shima Liu, Hongzhen Peng, Qingnuan Li, Liqiang Luo, Min Lv

**Affiliations:** ^1^ College of Sciences Shanghai University Shanghai China; ^2^ Division of Physical Biology & Bioimaging Center, Shanghai Synchrotron Radiation Facility, CAS Key Laboratory of Interfacial Physics and Technology, Shanghai Institute of Applied Physics Chinese Academy of Sciences Shanghai China; ^3^ University of Chinese Academy of Sciences Beijing China; ^4^ Key Lab of Health Technology Assessment (National Health Commission), School of Public Health Fudan University Shanghai China; ^5^ Shanghai Advanced Research Institute Chinese Academy of Sciences Shanghai China; ^6^ Shanghai Institute of Applied Physics Chinese Academy of Sciences Shanghai China

**Keywords:** biofilm formation, biofilm structure, concentration‐dependent, silver nanoparticles

## Abstract

**Objectives:**

To investigate the impact of silver nanoparticles (AgNPs) on the biofilm growth and architecture.

**Materials and methods:**

Silver nitrate was reduced by d‐maltose to prepare AgNPs in the presence of ammonia and sodium hydroxide. The physicochemical properties of AgNPs were characterized by transmission electron microscopy, ultraviolet‐visible spectroscopy and inductively coupled plasma mass spectrometry. The development of biofilm with and without AgNPs was explored by crystal violet stain. The structures of mature biofilm were visually studied by confocal laser scanning microscopy and scanning electron microscopy. Bacterial cell, polysaccharide and protein within biofilm were assessed quantitatively by colony‐counting method, phenol‐sulphuric acid method and Bradford assay, respectively.

**Results:**

The spherical AgNPs (about 30 nm) were successfully synthesized. The effect of AgNPs on *Pseudomonas aeruginosa* biofilm development was concentration‐dependent. Biofilm was more resistant to AgNPs than planktonic cells. Low doses of AgNPs exposure remarkably delayed the growth cycle of biofilm, whereas high concentration (18 μg/mL) of AgNPs fully prevented biofilm development. The analysis of biofilm architecture at the mature stage demonstrated that AgNPs exposure at all concentration led to significant decrease of cell viability within treated biofilms. However, sublethal doses of AgNPs increased the production of both polysaccharide and protein compared to control, which significantly changed the biofilm structure.

**Conclusions:**

AgNPs exert concentration‐dependent influences on biofilm development and structure, which provides new insight into the role of concentration played in the interaction between antibacterial nanoparticles and biofilm, especially, an ignored sublethal concentration associated with potential unintended consequences.

## 1 INTRODUCTION

Antimicrobial resistance (AMR) has gained considerable attentions due to its serious threat for public health and environmental safety.^1^, ^2^ Biofilm is considered to be the most important cause of bacterial resistance except for well‐known super‐bacteria induced by antibiotics abuse.^3^, ^4^, ^5^ Biofilm is the surface‐associated bacterial community integrated by microbial cells and self‐secreted extracellular polymeric substances (EPS),^6^, ^7^, ^8^ showing 10‐1000 times more resistant to traditional bactericides (eg, antibiotics and heavy metal ions) than planktonic cells.^9^, ^10^ This ubiquitous AMR system is extremely difficult to eliminate in clinic, industry and environment, which has given rise to serious infection and economic loss.^1^, ^11^ To address the urgent problem, many efforts have been made to design and fabricate various novel bactericides.^12^, ^13^ Recently, nanomaterials have been broadly applied in medicine, industry and environment due to their unique physicochemical properties, strong bactericidal activities and specific mechanisms including physical damage, oxidative stress, as well as photothermic destroy.^5^, ^14^, ^15^, ^16^ For example, most metal‐based nanomaterials can generate reactive oxide stress (ROS) to inactivate bacteria, such as titanium dioxide nanoparticles,^17^ cupric oxide nanoparticles^18^ and silver nanoparticles (AgNPs).^19^, ^20^ Release of metal ions from these nanoparticles also triggers the death of bacterial pathogens.^18^ Besides, some of them with specific optical and thermal properties can be exploited to inhibit the bacterial growth by selective non‐invasive photothermic destroy.^5^ These characteristics of nanomaterials different from conventional antimicrobials provide new insights into the prevention of biofilm formation and even eradication of formed biofilm.^21^

Silver nanoparticles hold a promising biomedical application because of relatively low manufacturing cost, excellent biocidal impact on a broad range of bacteria and probably lower inclination to cause bacterial resistance as compared to antibiotic.^22^, ^23^ AgNPs have been demonstrated that it could not only remarkably inhibit and kill planktonic bacteria,^19^ but also effectively prevent biofilm formation and destroy the biofilm architecture.^24^, ^25^, ^26^ However, single Ag nanoparticle tends to aggregate owing to its high surface energy resulted from large specific surface area.^27^, ^28^ To overcome the drawback, various AgNPs‐based composites are fabricated such as graphene oxide(GO)‐AgNPs and magnetic nanoparticles(MNP)@AgNPs, which can improve the stability of AgNPs, and provide synergistic antibacterial and anti‐biofilm effect superior to single AgNPs.^27^, ^29^ Notably, most of these studies conducted with the final inhibition efficacy of AgNPs, namely, how to obtain the optimal lethal concentration that is toxic to microorganisms but safe for human and environment. Yet, the effects of sublethal concentration of AgNPs exposure on biofilm are poorly understood, which is essential to understand the impacts of AgNPs released from commercial products on microbial ecosystems in the environment or engineered systems.^30^, ^31^ In addition, the biofilm formation is a complex serial process involving surface‐attached planktonic bacteria in the initial stage,^32^, ^33^ the attached cells proliferation and EPS generation, and the mature biofilm with maximum biomass.^7^, ^27^ No reports are available in the dynamic of biofilm growth exposed to AgNPs.

In this study, we employed *Pseudomonas aeruginosa* to examine biofilm development and structure responding to the different concentrations of AgNPs. *Pseudomonas aeruginosa* is a common model, Gram‐negative bacteria, broadly studied as a potential opportunistic human pathogen,^27^, ^30^ which has become a paradigm bacterium for biofilm research in the laboratory.^34^ The dynamic of *P aeruginosa* biofilm formation from planktonic to mature biofilm exposed to AgNPs at various concentrations was monitored by crystal violet (CV) stain. Furthermore, the biofilm architecture involving live bacteria, protein and glucose contents at the mature stage was explored in detail by visible images and chemical analysis. Our work might give a comprehensive picture of the interaction between antimicrobial nanomaterials and biofilm.

## 2 MATERIALS AND METHODS

### 2.1 Materials

Chemical agents (eg, silver nitrate, ammonia (28%‐30% w/w), sodium hydroxide, d‐maltose, phenol and sulphuric acid) and nutrient broth medium (NBM) were purchased from Sinopharm Chemical Reagent. Bradford Protein Assay Kit was purchased from Takara. Crystal violet (CV) was obtained from Sigma‐Aldrich (St. Louis, MO, USA). LIVE/DEAD Baclight Bacterial Viability Kit (L13152, Molecular Probes) and concanavalin A‐Alexa Fluor 647 conjugate were purchased from Invitrogen (USA). The ultrapure water was acquired from the Milli‐Q Integral Water Purification System.

The model bacterial strain *Pseudomonas aeruginosa* CCM 3955 (*P aeruginosa*) was obtained from the China General Microbiological Culture Collection Center (Institute of Microbiology, Chinese Academy of Sciences, Beijing). All microorganisms were stored at −80°C.

### 2.2 Preparation and characterization of AgNPs

Silver nanoparticles were synthesized according to the modified Tollens' process reported by Panacek et al^35^, ^36^ Briefly, silver nitrate (AgNO_3_, 1 mmol/L) was reduced by d‐maltose (10 mmol/L) in the presence of ammonia (5 mmol/L) and sodium hydroxide (9.6 mmol/L). Transmission electron microscopy (TEM, Tecnai G2 F20 S‐TWIN, FEI, USA) was used to observe the morphology of AgNPs. Ultraviolet‐visible (UV‐vis) spectroscopy (Agilent Cary 100, USA) was performed to record the surface plasmon peak of particles. The content of Ag element in synthesized AgNPs was measured by inductively coupled plasma mass spectrometry (ICP‐MS, Thermo Elemental X Series).

### 2.3 Biofilm formation

*Pseudomonas aeruginosa* was cultured in NBM at 37°C with shaking overnight. The bacterial cells were diluted to 0.01 of optical density (OD_600_ = 0.01, approximately 10^7^ CFU/mL) with fresh Nutrient broth and added to 24‐well plate covered with sterile coverslips (diameter 12 mm); then, AgNPs solution was added to a final concentration of 2, 6, 8 and 12 μg/mL, respectively. The plate was statically incubated at 37°C to form biofilm up to 24 hours. The biomass was quantified through CV stain every 2 hours. After incubation, the planktonic cells were discarded and the coverslip‐adhered biofilms were rinsed with phosphate‐buffered saline (PBS) three times to remove unbound cells and air‐dried for subsequent various analyses.

### 2.4 Crystal violet stain

To quantify the biomass of biofilm, they were stained with 0.1% (w/v) crystal violet for 15 minutes. The coverslips were gently washed three times with PBS after removing the excess stain. Digital camera (Canon EOS 750D) was employed to record the stained biofilm. Then, 1 mL of 95% ethanol was added to dissolve biofilm‐bound CV, whose absorbance was measured at 595 nm using a microtiter plate reader (Bio‐Rad 680, USA). All measurements were done at least three times.

### 2.5 Confocal laser scanning microscopy (CLSM)

Biofilm three‐dimensional (3D) structure was observed by CLSM and quantified using comstat according to our previous work.^27^ Briefly, SYTO 9 (488/500 nm) and concanavalin A‐Alexa Fluor 647 (647/668 nm) probe were used for labelling live cells and EPS, respectively. Confocal images were acquired in the same field of view using Leica SP8 upright multiphoton laser scanning microscope with a 63 × oil immersion objective. The confocal images were analysed using software for simultaneous visualization and quantification of EPS and bacterial cells within intact biofilms, while comstat was used for quantitative analysis.^27^, ^37^, ^38^

### 2.6 Scanning electron microscopy (SEM)

The morphology of biofilms was analysed by SEM. Prepared biofilms were fixed with 2% glutaraldehyde for 2 hours at 4°C, and then dehydrated via freeze‐drying for 24 hours. The samples were coated with gold and observed by SEM (FEI, Magellan 400, USA) with a TLD detector in SE mode and accelerating voltage of 10 kV.

### 2.7 EPS assay

Protein and polysaccharide in EPS were measured by Bradford assay and phenol‐sulphuric acid method, respectively. Two methods were briefly described as follows.

Phenol‐sulphuric acid method: Harvested biofilm was suspended in 1 mL ultrapure water through ultrasonic oscillation. Next, the biofilm suspension was centrifuged (10 000 rpm, 2 minutes) to acquire supernatant solution to which equal volumes of 5% phenol and five volumes of concentrated H_2_SO_4_ were added. The mixture was heated at 90°C in a water bath for 15 minutes and cooled at room temperature for 15 minutes before measuring the absorbance at 490 nm.^39^, ^40^

Bradford assay: The supernatant solution was obtained according to the same process in polysaccharide quantification. Then, equal volumes of supernatant solution and Bradford Dye Reagent were mixed and reacted at room temperature for 5 minutes before measuring the absorbance at 595 nm.

Amount of the polysaccharide and protein were both determined directly through the standard curve (Figure S1).

### 2.8 Statistical analysis

All data were expressed as mean plus or minus standard deviation (±SD). Student's *t* test was used to evaluate the statistically significant differences between groups. All assays were done at least three times.

## 3 RESULTS

### 3.1 Characterization of synthesized AgNPs

**Figure 1:**
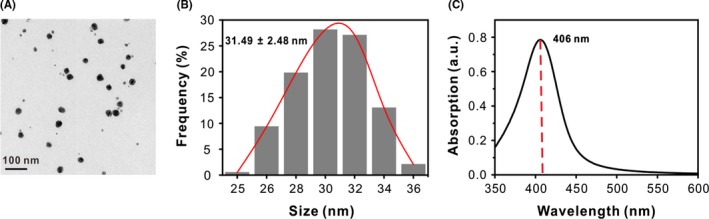
Characterization of the synthesized AgNPs. A, Typical TEM images and (B) size distribution of AgNPs. C, UV‐vis spectrum of AgNPs

Silver nanoparticles were prepared by d‐maltose reduction of AgNO_3_ in the presence of ammonia and sodium hydroxide according to Tollens' method.^35^, ^36^ d‐maltose is a mild reductant, which reduced the complex cation [Ag(NH_3_)_2_]^+^ to AgNPs. The transmission electron microscopic (TEM) images represented a relatively uniform dispersion of spherical Ag nanoparticles (Figure 1A). The narrow size distribution of AgNPs was 31.49 ± 2.48 nm (Figure 1B), which was randomly measured 195 spherical‐shaped particles in the TEM images. The UV‐Vis spectrum revealed a characteristic surface plasmon absorption peak at 406 nm (Figure 1C), clearly indicating the successful formation of AgNPs.^22^, ^27^ Dynamic light scattering (DLS) showed that particles were negatively charged with a zeta potential of −23.8 mV and hydrodynamic diameter of 37.8 nm (Figure S2), which suggested that the synthesized AgNPs were stable in the water.

### 3.2 Dynamic growth of biofilm exposed to AgNPs

**Figure 2:**
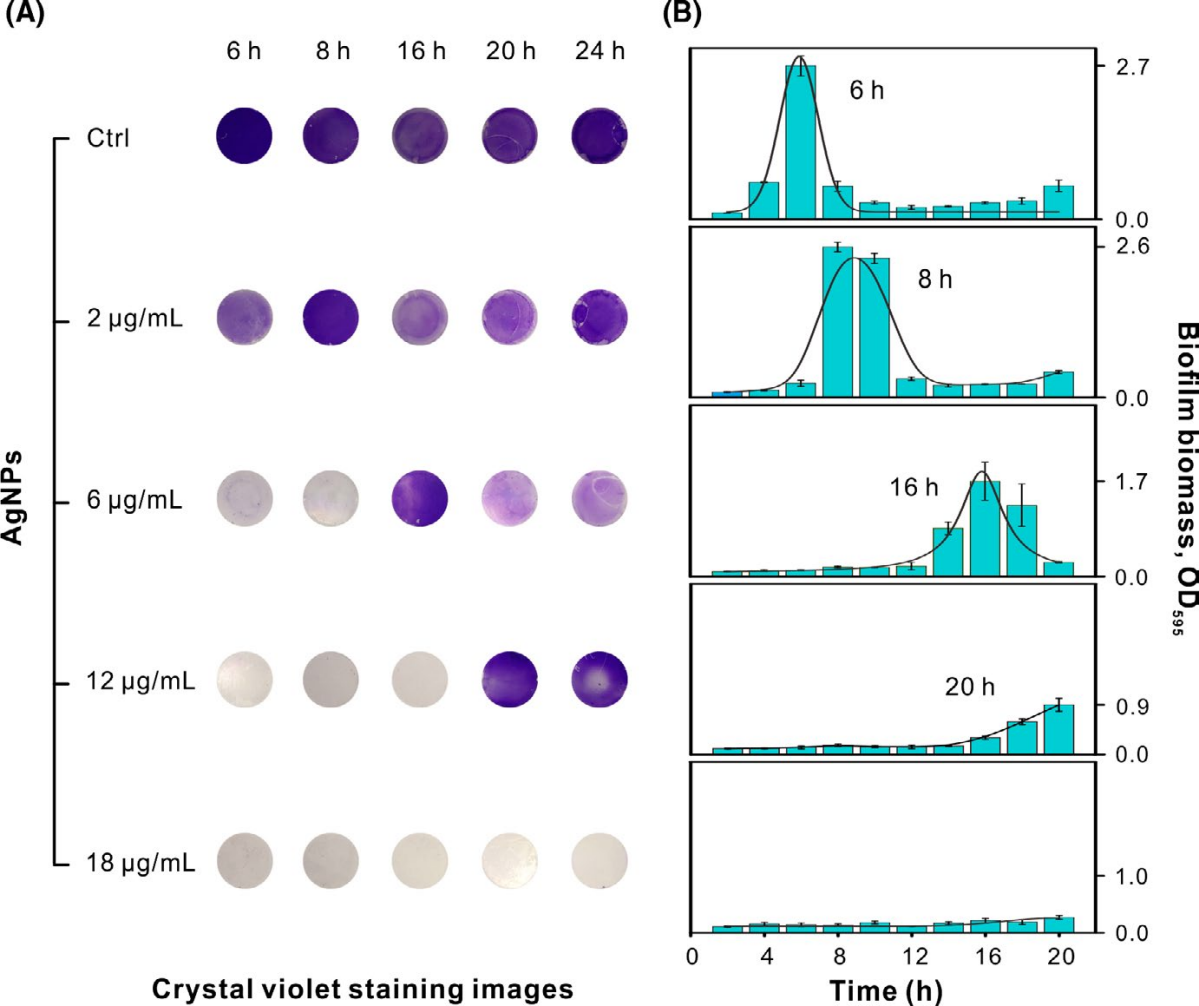
Dynamic of biofilm growth in environment with and without AgNPs. A, Digital images of biofilms stained by CV at some point. B, Dynamic of biofilm formation. The concentration of AgNPs was 0, 2, 6, 12 and 18 μg/mL, respectively

To confirm the antibacterial properties in vitro, turbidity test was used to study the antimicrobial property of AgNPs against *P aeruginosa*. The results found AgNPs exhibited considerable antibacterial capacity, wherein the minimum inhibitory concentration (MIC) value of AgNPs was 2 μg/mL against *P aeruginosa* (Figure S3). To quantify the dynamic of biofilm growth in the presence of AgNPs, we observed the biofilm development for periods of up to 24 hours. Biofilm formation begins with the attachment of planktonic cells on the substrate. Following, adhesion bacteria grow and largely secrete EPS to mature.^41^, ^42^ The process ends with single cell dispersal from the mature biofilm, which means the start of the new biofilm growth.^27^, ^43^ As shown in Figure 2, CV stain images and the absorbance at 595 nm indicated *P aeruginosa* biofilm exhibited AgNPs resistance properties, and the biofilm growth was completely inhibited when the concentration reached 18 μg/mL *P aeruginosa* biofilm treated with sublethal concentrations of AgNPs went through the same growth stage but different time compared to the untreated sample. The normal planktonic *P aeruginosa* cell attached to the coverslip and established mature biofilm 3D architecture after 2 and 6 hours, respectively. The cell proliferation and biofilm maturation of floating cells exposed to 2, 6 and 12 μg/mL, respectively, delayed to 8, 16 and 20 hours (Figure 2B). Interestingly, the biomass of mature biofilm (8 hours) in the presence of the MIC of AgNPs against planktonic *P aeruginosa* (2 μg/mL) was closer to control sample (6 hours). However, the maximum biomass of biofilms showed dose‐dependent decrease when the concentration of AgNPs exposure ranges from 2 to 18 μg/mL. These findings demonstrated *P aeruginosa* cells lost their ability to develop biofilm in a concentration‐dependent manner when they were exposed to AgNPs at the concentration higher than MIC.

### 3.3 Morphology and structure of mature biofilms

**Figure 3:**
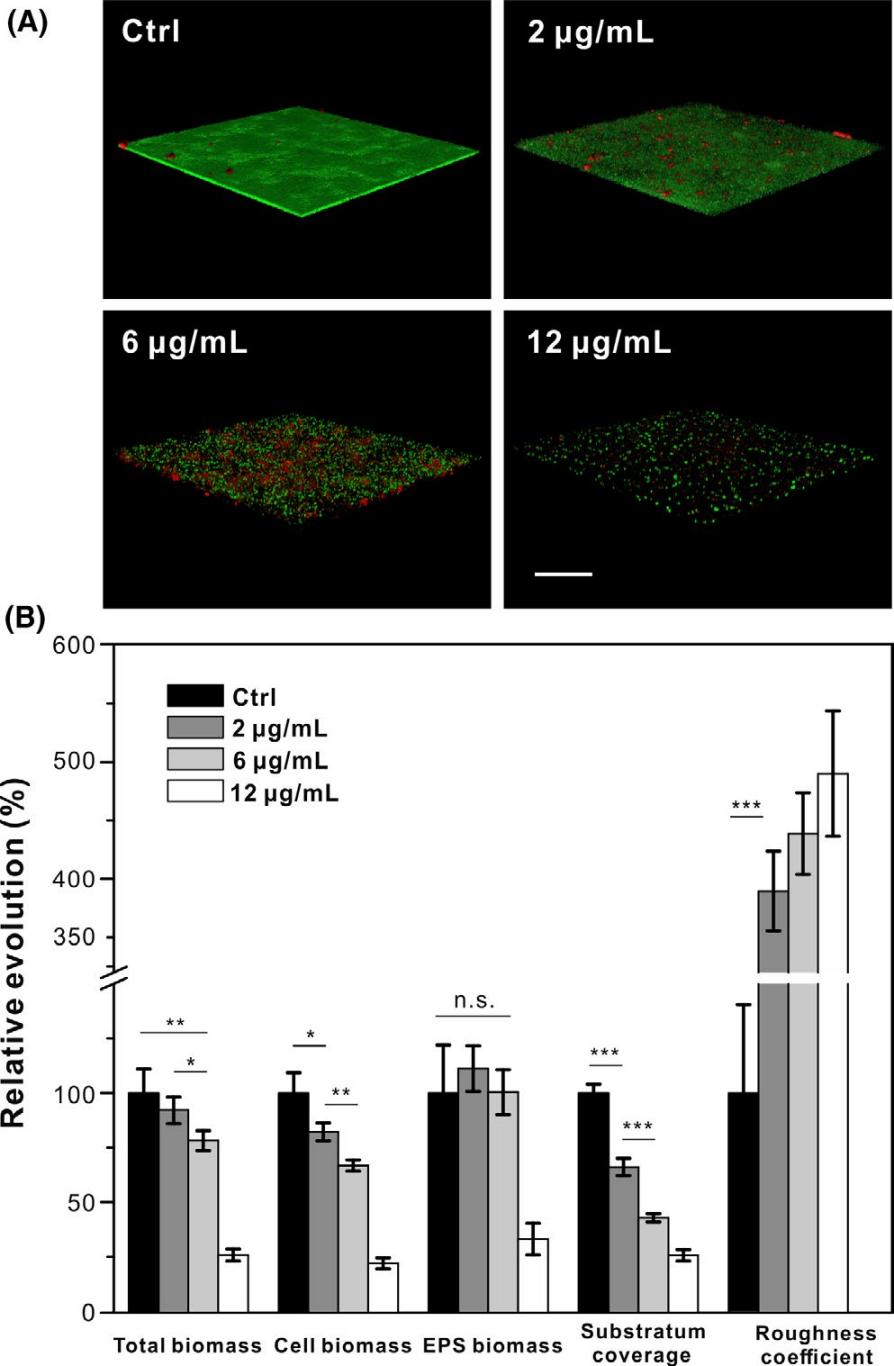
Confocal images and quantification of the mature biofilms exposed to AgNPs. A, Confocal 3D images of biofilms. Bacterial cells stained with SYTO9 dye (green) and EPS stained with Con A‐Alexa Fluor (red). Scale bar, 50 μm. B, Quantitative characterization of the biofilm morphology by comstat software based on fluorescent images. * indicates the significant difference at *P* < 0.05, **P < 0.01, ***P < 0.001; ns non‐significant

To further investigate biofilm architecture at the mature stage, bacteria and EPS components within 3D biofilm were explored by multiphoton CLSM. SYTO 9 probe with green fluorescence was used for labelling bacterial cells while concanavalin A‐Alexa Fluor 647 with red fluorescence was used to visualize EPS.^27^, ^38^ Figure 3A reveals the dose‐dependent influence of AgNPs on the mature biofilm morphology. Tight and smooth structure of biofilm with 2 μg/mL AgNPs was observed, which is similar to control sample. However, the biofilm was visibly decreased along with the increase in exposure concentration of AgNPs. comstat analysis based on 3D biofilm images was shown in Figure 3B, which revealed the concentration‐dependent effect of AgNPs towards biofilm morphology. A decrease in total biomass, bacterial biomass, and substratum coverage and an increase in roughness coefficient with increasing concentrations of NPs were gained. Total biomass of treated biofilm was 8.95 ± 0.98, 8.24 ± 0.54, 7.00 ± 0.40 and 2.31 ± 0.25 μm^3^/μm^2^, respectively, when it was exposed to various concentrations of AgNPs (0, 2, 6 and 12 μg/mL). EPS biomass in biofilms treated with 2 μg/mL AgNPs was slightly higher than that in other groups, but no significant difference was observed among all groups. More EPS secreted may be due to a passive survival strategy activated by stress conditions.^44^ Bacterial biomass was reduced to 4.92 ± 0.24, 4.00 ± 0.15 and 1.32 ± 0.15 μm^3^/μm^2^ after treatment of AgNPs (2, 6 and 12 μg/mL), which resulted from the toxicity of AgNPs to bacterial cells. The substratum coverage of biofilms with AgNPs (0, 2, 6 and 12 μg/mL) was 95.79% ± 3.80%, 63.33% ± 3.73%, 41.07% ± 1.80% and 24.61% ± 2.46%, respectively. Discrete bacterial colonies caused by AgNPs significantly increased the roughness coefficient of biofilm. The altered action of the roughness coefficient was notably characterized at 2 μg/mL AgNPs compared to the non‐exposed biofilms. Thus, these results demonstrated that the decreasing number of surface‐adhesion bacteria weakened the biofilm formation.

**Figure 4:**
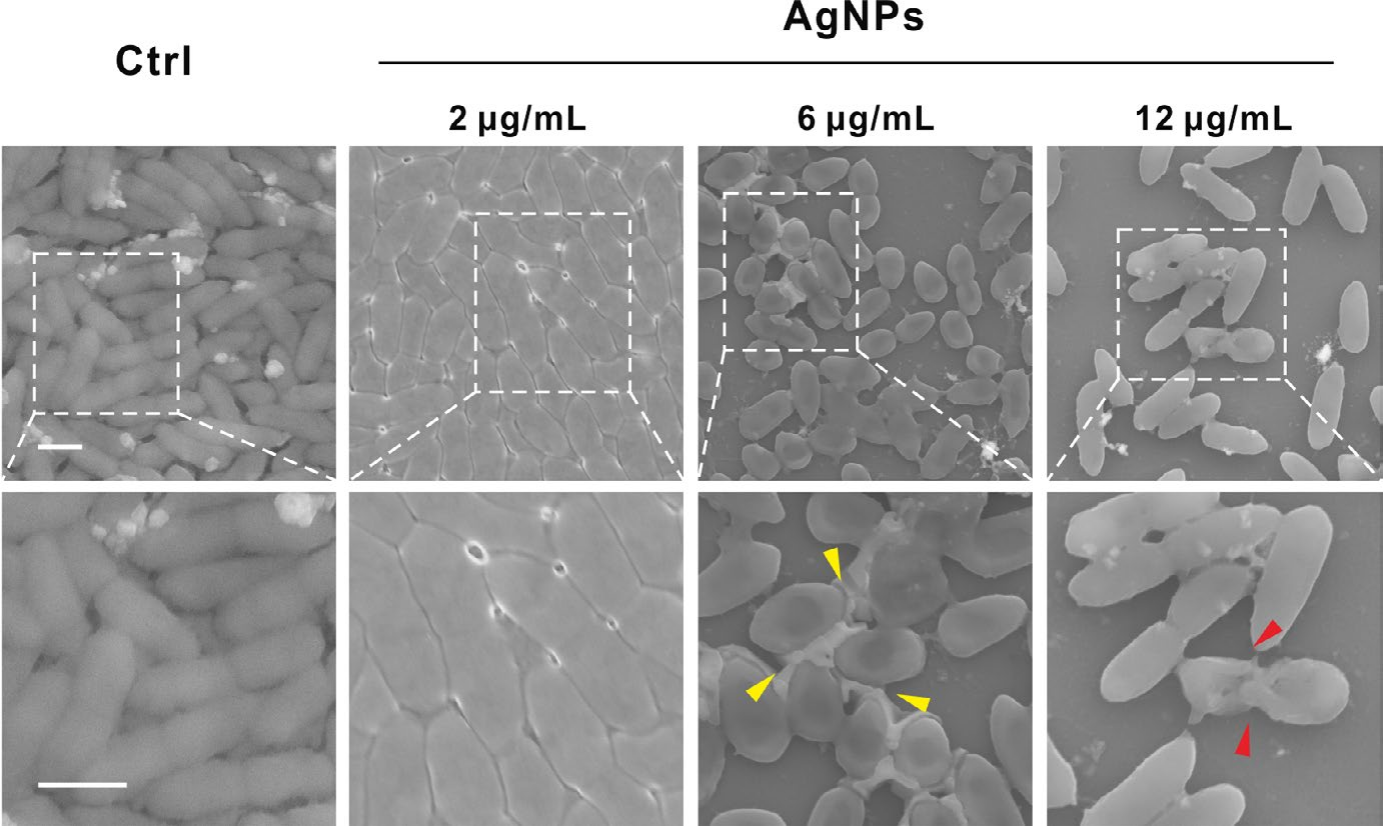
SEM images of mature biofilm exposed to AgNPs. The second panel is high‐magnification images of the indicated portion in the first panel. Typical structures of biofilms contain bacterial cells and EPS, which are, respectively, indicated with red and yellow arrows. Scale bar, 1 μm

Scanning electron microscopy (SEM) was performed to observe refined morphology of biofilm, which was consistent with confocal results. *Pseudomonas aeruginosa* biofilm exposed to 2 μg/mL AgNPs was similar to control biofilm, showing fully covered compact structure and integrated morphology (Figure 4). However, when the concentration of AgNPs reached 6 μg/mL, bacterial reduction and distinct EPS‐matrix formation surrounding the bacterial strains (yellow arrows) could be observed. High concentration of AgNPs exposure (12 μg/mL) could lead to the disruption of the bacterial cell membrane (red arrows), which agreed with previous work that Ag nanoparticles inactivated bacteria by damaging cellular integrity.^19^, ^22^

**Figure 5:**
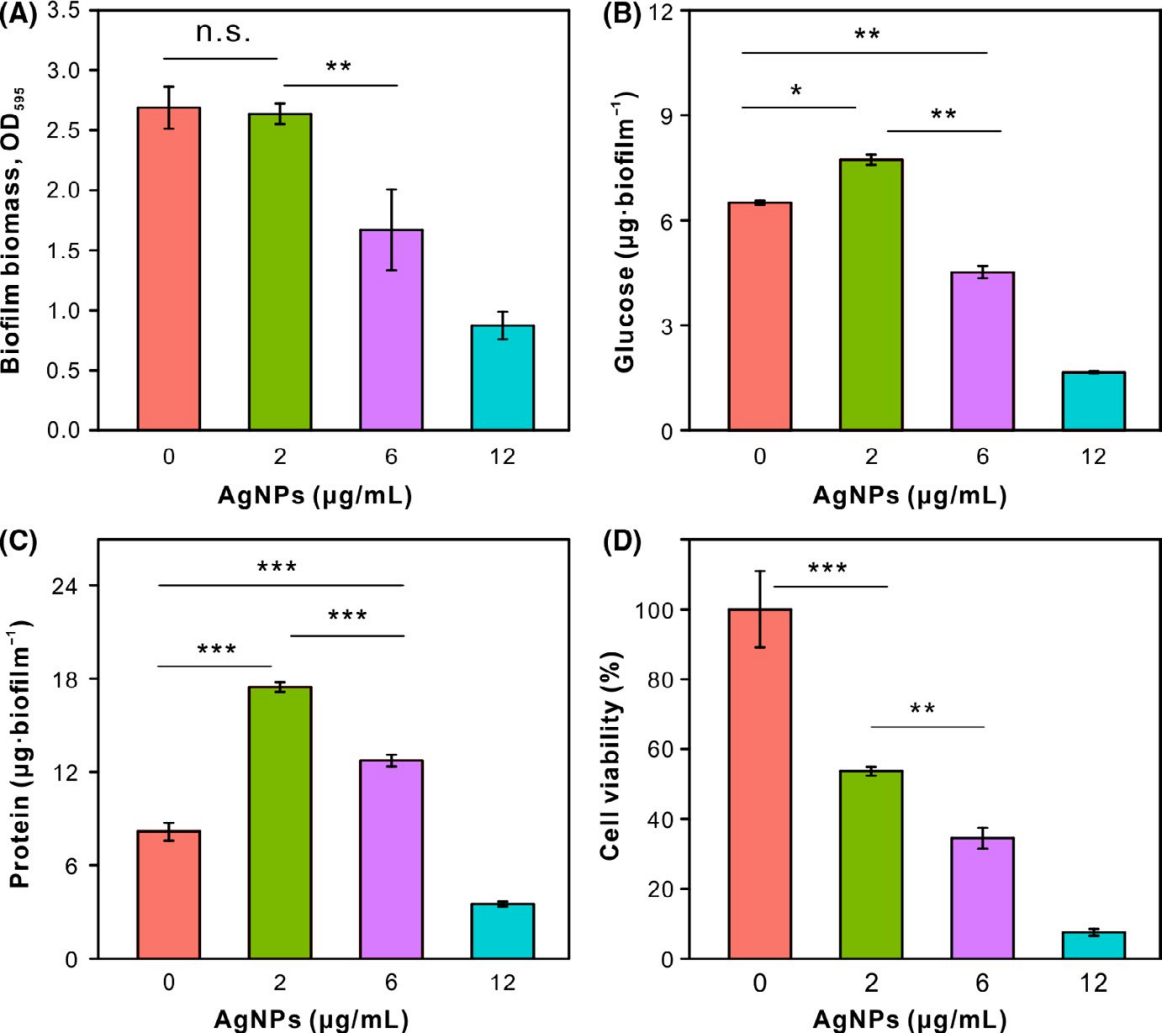
Compositions of mature biofilms with and without AgNPs. A, Analysis of total biomass by CV stain. B, Quantification of polysaccharide and (C) protein within biofilms by chemical approaches. D, Viable cells in biofilms were determined by colony‐forming unit assay. * indicates the significant difference at *P* < 0.05, **P < 0.01, ***P < 0.001; ns non‐significant

As we know, EPS is a multicomponent complex containing polysaccharide, protein, extracellular DNA (eDNA) and so on.^6^, ^27^ Among various components, protein is used for bacterial adhesion to surfaces,^45^ and a polysaccharide also contributes to the development and structure of biofilm.^46^, ^47^ To further explore mature biofilm architecture, the EPS components including polysaccharide and protein were determined by chemical quantification while the number of viable cells was estimated by colony‐counting method. As shown in Figures 2B and 5A, total biomass detected by CV stain including biomass of live and dead bacteria was reduced by 5.69%, 37.87% and 67.52%, respectively, after exposure to AgNPs (2, 6 and 12 μg/mL). The inhibition of bacterial growth was observed when exposed to AgNPs (Figure S4). As shown in Figure 5D, the viability of bacteria in treated biofilm (2, 6 and 12 μg/mL) was reduced by 46.28%, 65.50% and 92.43%. These results suggested that AgNPs is highly toxic to bacteria. The concentration of 2 μg/mL AgNPs stress resulted in the secretion of more polysaccharide and protein than those within the control group (Figure 5B,C), which was consistent with the confocal imaging result (Figure 3B). Although less polysaccharide was secreted within biofilm treated with 6 μg/mL AgNPs than that in the control group, this could be contributed to less “EPS producers” because of the growth‐inhibition effect of AgNPs and lower cell viability.

## 4 DISCUSSION

Biofilms are microbial communities typically encased in a self‐produced EPS, resulting in the serious AMR.^6^ Microorganisms that reside in biofilm the invasion of antibiotics or other antimicrobial agents.^4^, ^43^ Although the antibacterial nanoparticles are considered as promising candidates for addressing AMR,^19^, ^48^ multiple cycles of treatment below lethal concentration also led to gradual increases in MIC of AgNPs,^36^ which exacerbates our concerns about whether biofilm exhibits tolerance and resistance to it. In this study, the results showed that AgNPs possessed the strong dose‐dependent anti‐biofilm capacities, which conformed to previous work.^19^, ^22^ It is worth noting that *P aeruginosa* biofilm became more resistant to AgNPs. The inhibition concentration of AgNPs against biofilm (18 μg/mL) was nine times higher than planktonic cells (2 μg/mL), which is very similar to the behaviour of biofilm in the presence of antibiotics.^9^, ^10^ The strong prevention efficacy of AgNPs was explained by several hypotheses, (a) the accumulation of Ag nanoparticles on bacterial surface can alter the permeability of the cell membrane, thus cause the cytoplasm leakage and cell death, (b) ROS generation by AgNPs can disrupt the cellular integrity and even damage intracellular DNA and metabolism, and (c) the release of Ag ions from AgNPs is also toxic to bacteria.^20^, ^27^ Actually, the MIC value of Ag^+^ against planktonic *P aeruginosa* is similar to AgNPs, but it has stronger anti‐biofilm properties than AgNPs (Figure S5). Thus, anti‐biofilm property of AgNPs may result from synergistic action of Ag ions and nanoparticles.

Upon low doses of AgNPs exposure, the low growth of bacteria and high secretion of EPS was the key strategy for bacteria to protect themselves from stress condition.^44^, ^48^ The higher but sublethal concentration of AgNPs led to longer lag phase for biofilm formation. Two main forms of tolerance are concluded, namely, “tolerance by slow growth” and “tolerance by lag phase.”^49^, ^50^ Tolerance by slow growth occurs at steady state, whereas tolerance by lag is a transient state that is induced by starvation or stress.^51^ In our experiment, the poor condition caused by the addition of AgNPs was the main reason for slow growth tolerance while “tolerance by lag phase” also resulted in the delay of growth recovery (Figure 2),^52^ thus caused a mixed phenotype of resistance and tolerance.^53^, ^54^ To verify whether the phenotype appeared, we detected the efficacy of AgNPs against bacterial indwelling treated biofilms. The MIC of AgNPs towards filial generation strains was all 4 μg/mL (Figure S6). The little higher concentration may be due to bacteria in a crowded condition of biofilm rather than AgNPs stress. Maybe one cycle was not enough to establish resistance. In addition, bacteria in biofilm secret more EPS (Figures 3 and 5), which provides a barrier for attached live bacteria on the surface against AgNPs attack. Besides, the aggregation of AgNPs may weaken their capacities combating bacterial biofilms.^27^, ^36^

## 5 CONCLUSION

In summary, this work reported the concentration‐dependent impact of AgNPs on the development of *P aeruginosa* biofilm, especially, an ignored potential unintended result associated with bacterial exposure to sublethal concentrations of AgNPs. The results showed that biofilm was more resistant to AgNPs than planktonic cells. Low concentrations of AgNPs exposure made the delayed growth of biofilm and enhanced polysaccharide and protein secretion in mature biofilm, suggesting the changed biofilm dynamic and architecture. Nevertheless, high concentration (18 μg/mL) of AgNPs completely prevented biofilm formation. This study is helpful for further understanding of the role of concentration in the interaction between antibacterial nanoparticles and biofilm.

## CONFLICT OF INTEREST

The authors declare there is no conflict of interest.

## AUTHOR CONTRIBUTIONS

Jingyang Guo and Min Lv conceived and designed the experiment. Jingyang Guo performed the experiments. Jingyang Guo, Simin Qin, Shima Liu and Min Lv analysed the data. Jingyang Guo and Min Lv co‐wrote the paper. All authors discussed the results, commented and agreed to publish the manuscript.
