# Supplementary material for: Silver nanoparticles exert concentration‐dependent influences on biofilm development and architecture
Source: Cell Prolif. 2019 May 3;52(4):e12616. doi: 10.1111/cpr.12616 (PMC6668980; doi:10.1111/cpr.12616)
Supplement: Supplementary file 1 [file CPR-52-e12616-s001.docx]

**Supporting Information**

**Silver nanoparticles exert concentration-dependent influences on biofilm development and architecture**

Jingyang Guo^1,2^, Simin Qin^2,4^, Yan Wei^5^, Shima Liu^1,2^, Hongzhen Peng^2,3^, Qingnuan Li^6^, Liqiang Luo^1*^, Min Lv^2,3*^

1. College of Sciences, Shanghai University, Shanghai 200444. P.R. China

2. Division of Physical Biology & Bioimaging Center, Shanghai Synchrotron Radiation Facility, CAS Key Laboratory of Interfacial Physics and Technology, Shanghai Institute of Applied Physics, Chinese Academy of Sciences, Shanghai 201800, P.R. China

3. Shanghai Advanced Research Institute, Chinese Academy of Sciences, Shanghai 201210, China.

4. University of Chinese Academy of Sciences, Beijing 10049, China

5. Key Lab of Health Technology Assessment (National Health Commission), School of Public Health, Fudan University, Shanghai 200032, China

6. Shanghai Institute of Applied Physics, Chinese Academy of Sciences, Shanghai 201800, China

Corresponding Authors: lvmin@sinap.ac.cn; luck@shu.edu.cn

**Methods**

**Dynamic light scattering (DLS)**

The average size and zeta potential of the synthesized AgNPs were determined by the dynamic light scattering and electrophoretic mobility methods (Malvern Zeta Sizer Nano ZS90, UK).

**Minimum inhibitory concentration (MIC) assay**

The process to determine the MIC was briefly showed. Bacteria at a concentration of 10^6^ CFU/mL were cultured in Nutrient broth in the presence of AgNPs (106 μg/mL of silver) at 37 °C at 220 rpm for 24 h. The final concentrations of added AgNPs were 0, 1, 2, 4, 8, and 12 μg/mL, respectively. The MIC of AgNPs was determined as the lowest silver concentration that inhibited visible growth of the tested microorganisms.


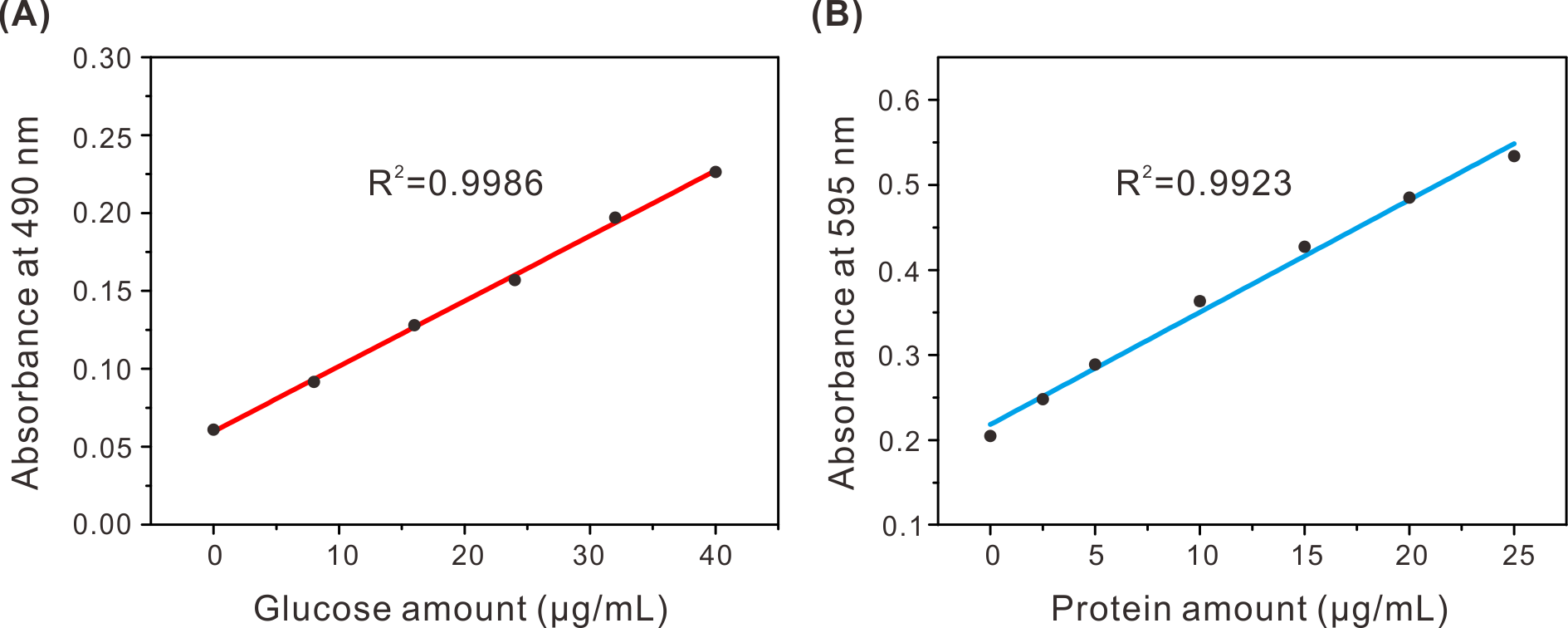


Figure S1 Standard curves for quantification analysis of polysaccharide (A) and (B) protein.


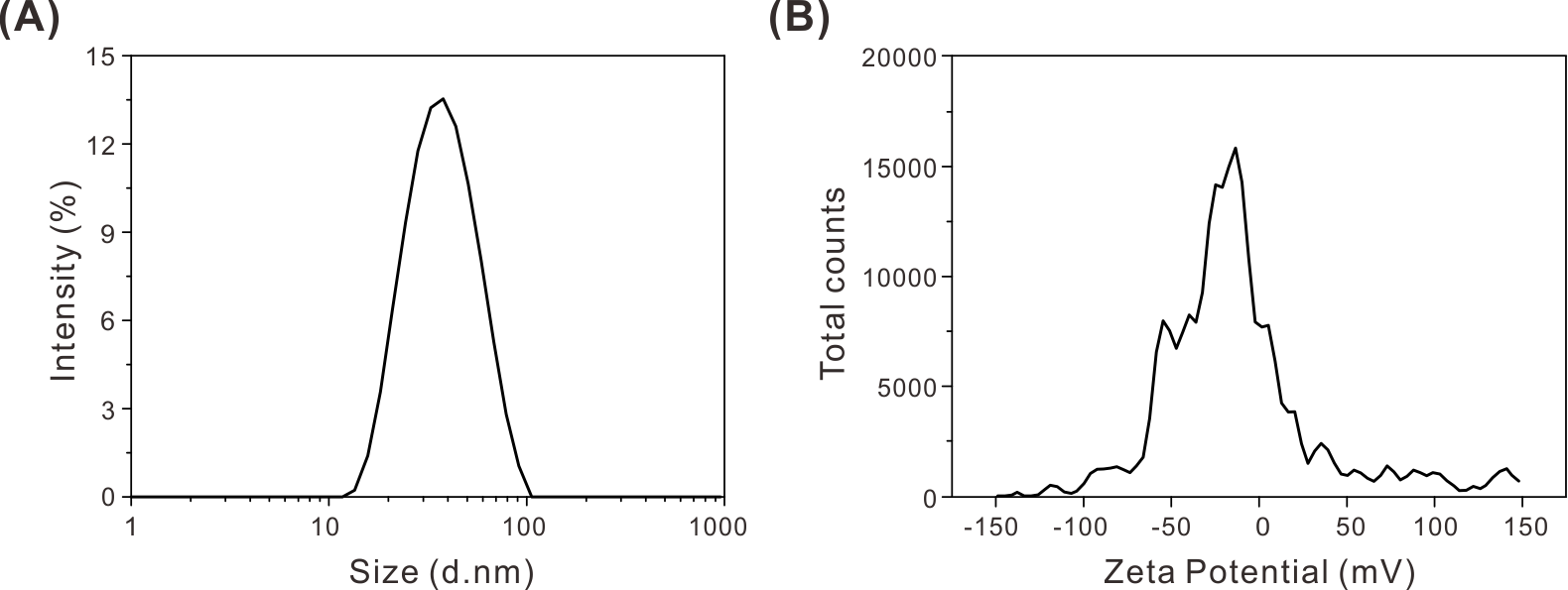


Figure S2 (A) Hydrodynamic diameter by DLS. (B) Zeta potential of AgNPs.


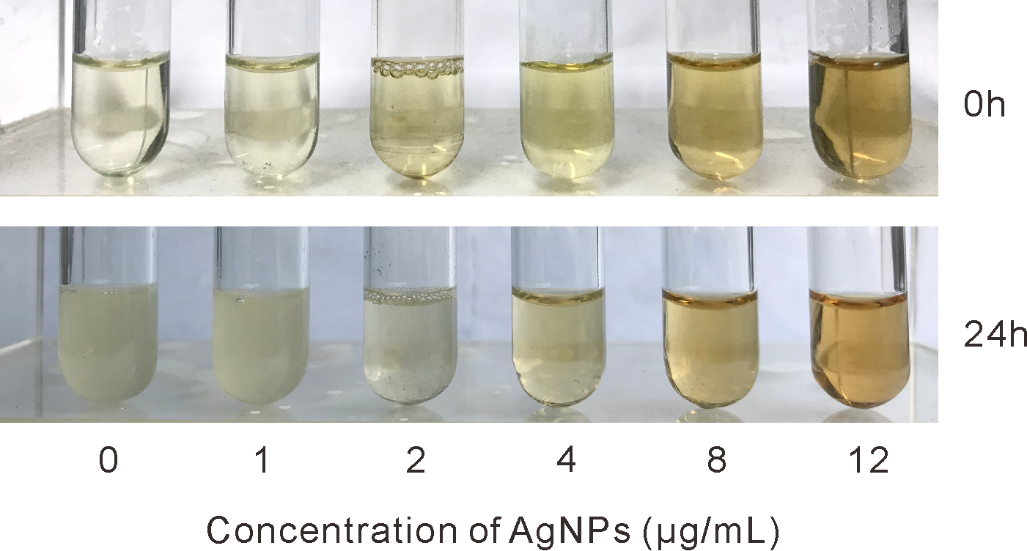


Figure S3 MIC of AgNPs against *P. aeruginosa* strain.


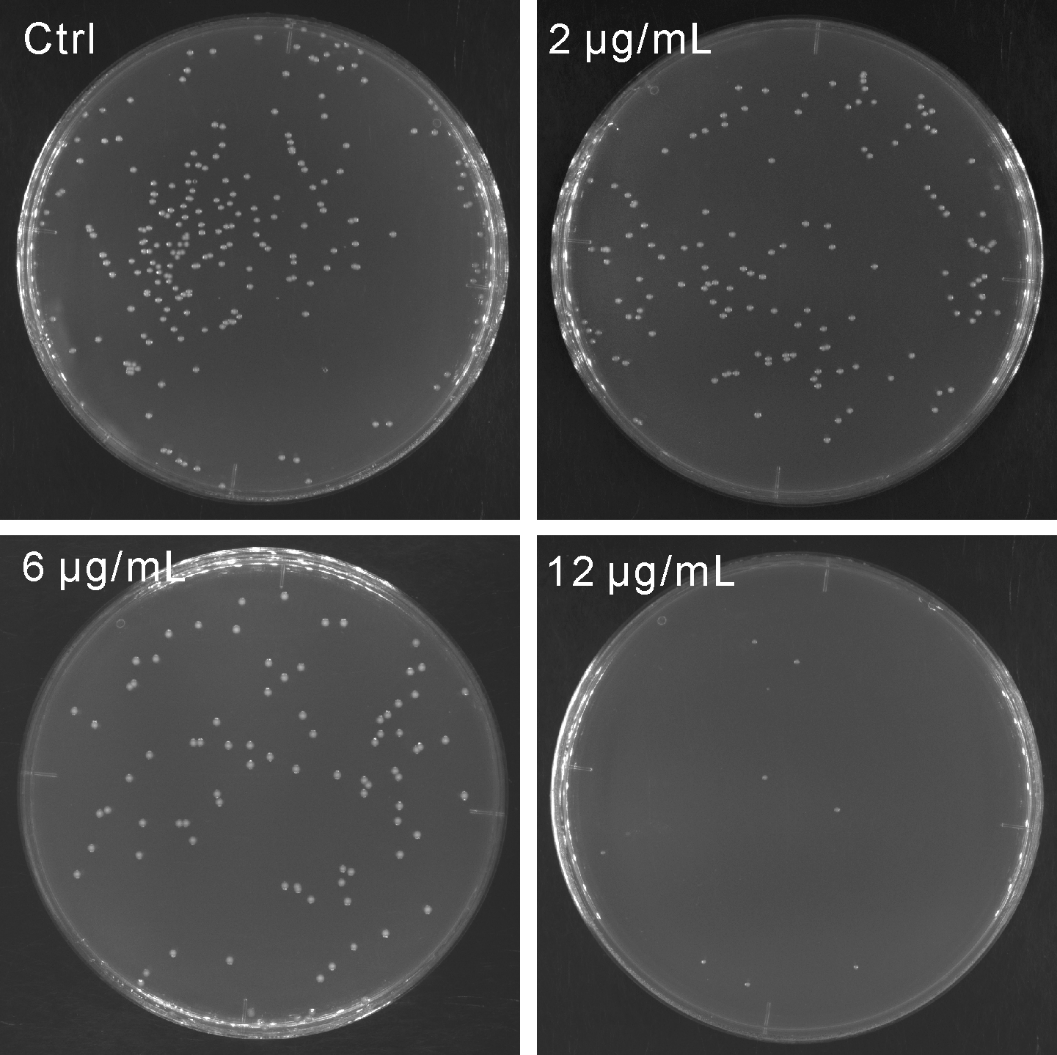


Figure S4 Bacteria cells within biofilms exposed to various concentrations of AgNPs.


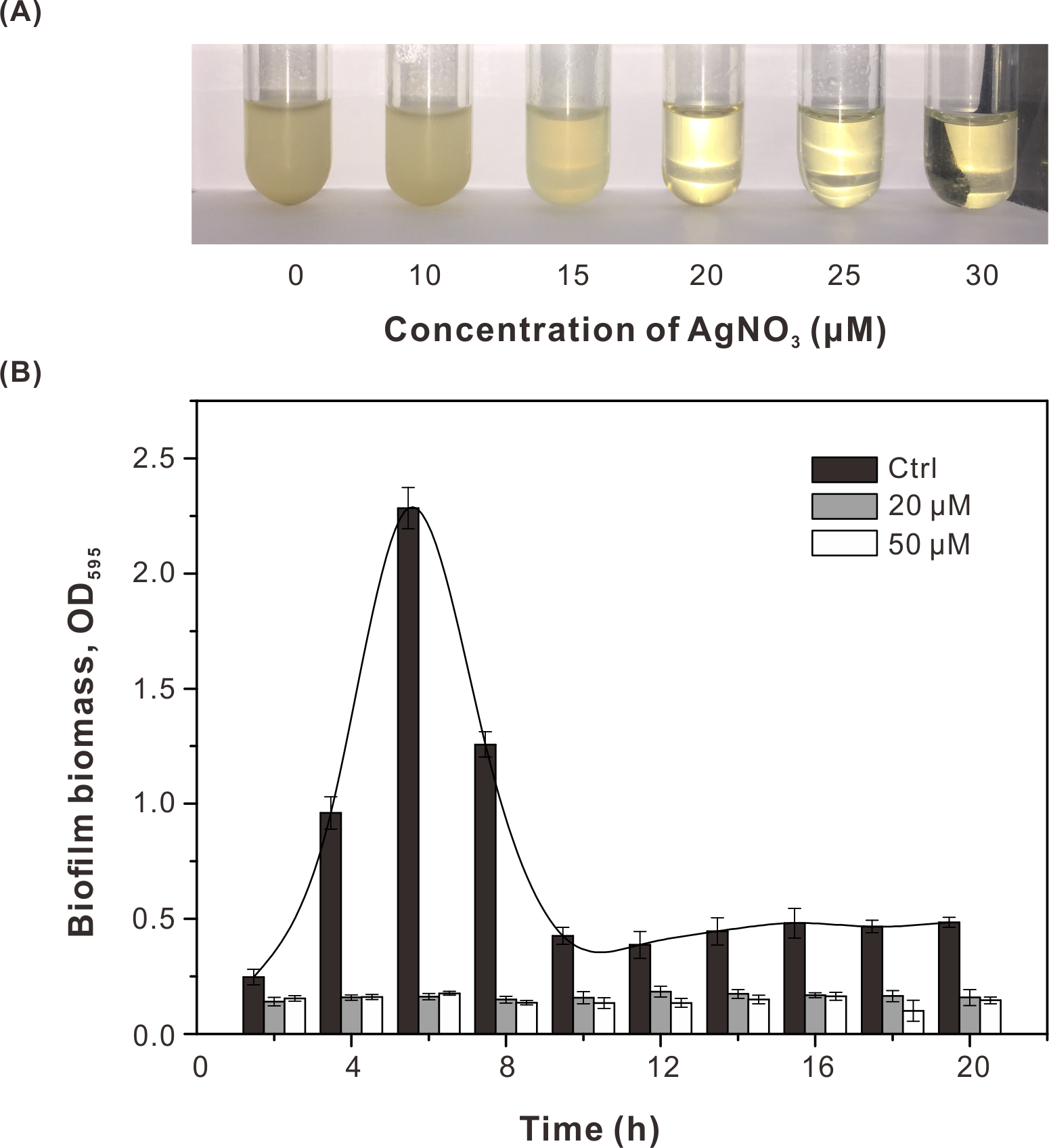


Figure S5 (A) MIC of Ag^+^ against *P. aeruginosa* strain. (B) Dynamic of biofilm formation exposed to Ag^+^ at the concentration of 0, 20, 50 μM, respectively.


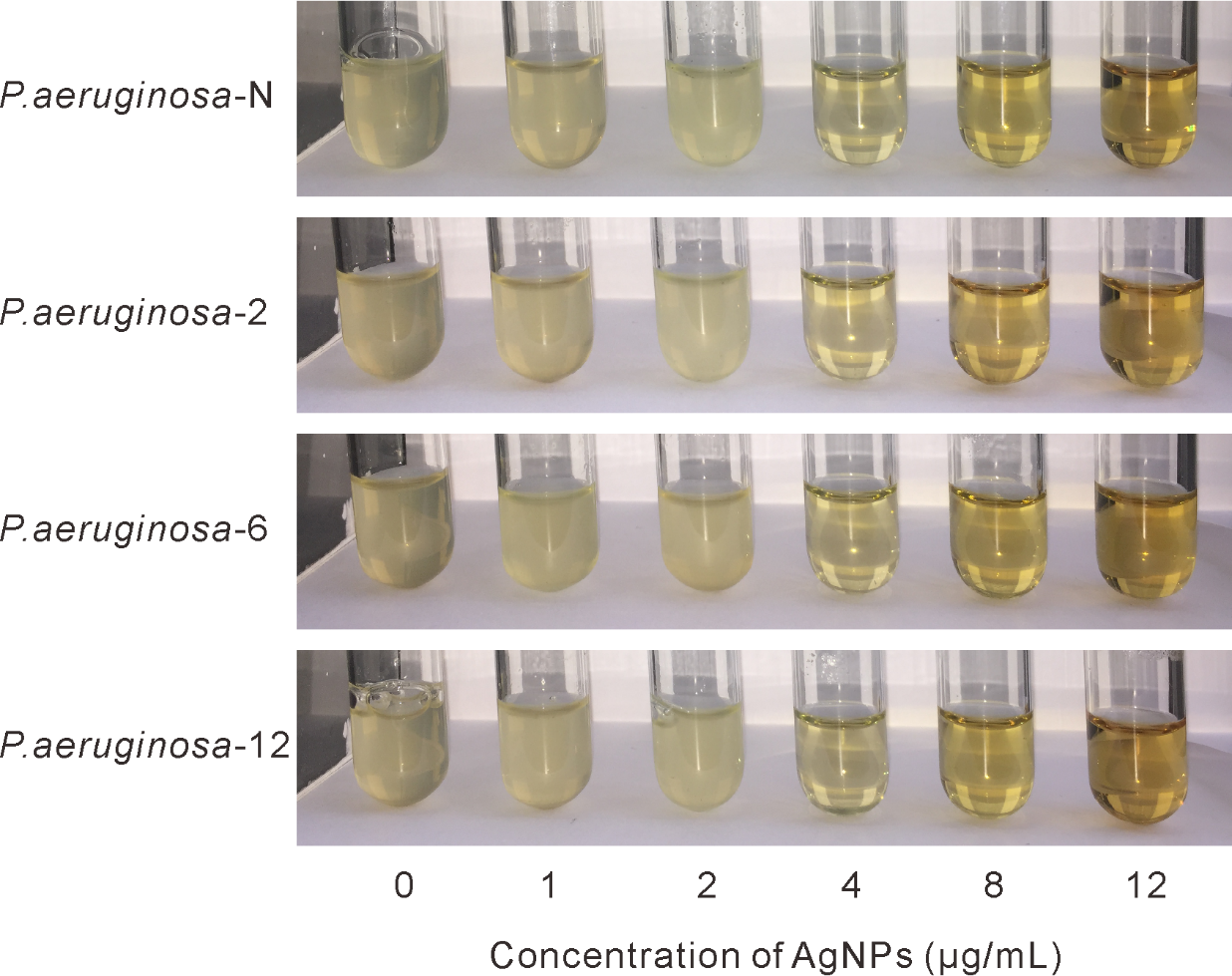


Figure S6 MIC of AgNPs against survival bacteria within biofilms. *P.aeruginosa*-N, *P.aeruginosa*-2, *P.aeruginosa*-6 and *P.aeruginosa*-12 respectively represents survival *P. aeruginosa* in mature biofilms treated with 0, 2, 6, and 12 μg/mL AgNPs.
